# Acid Properties of Hierarchical Zeolites Y

**DOI:** 10.3390/molecules25051044

**Published:** 2020-02-26

**Authors:** Mariusz Gackowski, Jerzy Datka

**Affiliations:** Jerzy Haber Institute of Catalysis and Surface Chemistry Polish Academy of Sciences, Niezapominajek 8, PL-30239 Krakow, Poland; ncgackow@cyf-kr.edu.pl

**Keywords:** mesoporous zeolites, acidity, desilication

## Abstract

The article reviews different strategies towards obtaining mesoporous zeolites Y: desilication; surfactant templating and assembly of zeolite crystals. The impact of those methods on physicochemical properties is covered, with a special focus on the acidity of the samples measured with infrared (IR) spectroscopy. The methods of characterization of acidity are presented. Quaternary ammonium cations used for desilication lead to obtaining crystalline; mesoporous and highly acidic zeolites. Si-OH-Al groups of extremely high acidity can be produced by calcination in a humid atmosphere. When the conditions are optimized, post-synthetic surfactant templating allows crystalline mesoporous zeolite to be obtained with no loss of material. All mesoporous zeolites Y proved to be active catalysts in liquid phase isomerization, catalytic cracking, and other reactions.

## 1. Introduction

Zeolites are well-known catalysts of high surface area, high hydrothermal and thermal stability, structural pores of molecular dimensions, and containing strong Brønsted acid sites. These unique properties are responsible for their application as catalysts in many major (petro)chemical processes, e.g., [1,2,3]. The fact that the active sites are situated inside the micropores is advantageous for the stabilization of carbocations, by negative charges in the framework, and shape selectivity in the case of some zeolite frameworks. However, diffusional limitations are frequently observed when large molecules are processed in large-crystal zeolites with narrow pores. Moreover, such mass transfer limitations may cause a fast catalyst deactivation [4].

To improve the catalytic effectiveness in chemical reactions, several approaches were proposed. One of them was the synthesis of extra-large pore zeolites [5,6]. Another one was the preparation of zeolite nanoparticles [7,8,9] or assembly of zeolitic micrograins producing micro-meso-macroporous structures [10,11]. A very effective way was the synthesis of hierarchical zeolites containing more than one level of pores. Carbon particles [12,13,14,15,16] or template micelles [17,18,19,20,21,22] were incorporated into zeolite crystals, during the synthesis, and these were next burned off producing the additional mesopores. This strategy is called “bottom up”. Apart from some examples of organic surfactants that produce crystalline material (like silane-terminated quaternary amines [17,18,19,22]), this approach often results in phase separation and amorphous material [23]. Mesoporous aluminosilicates can be produced as well by micelles-templating of zeolite seeds [24]. Zeolite crystals can be modified with organic surfactants without deterioration of their structure with the method called “mesostructuring” [23,25,26,27,28,29]. Another strategy called “top down” is postsynthesis treatment in which demetalation was found to be the most practical method for obtaining hierarchical zeolites. Dealumination is, in general, a less efficient method to create mesopores, while the desilication of zeolites in alkaline solutions turns out to be the most effective way to produce mesoporous zeolites.

Desilication has been applied to various zeolites such as: MOR [30,31,32], FER [33,34], BEA [35,36,37], MAZ [38] among others. Most desilication studies have been applied to MFI zeolites ([39,40,41,42,43,44,45,46,47,48], among many others) and zeolites possessing good porosity, acidity, and catalytic activity were obtained. The composition of MFI zeolites (Si/Al = 30–50) is optimal for the extraction of Si from the zeolite, moreover, the formation of mesopores in the middle pore zeolite improves significantly the transport of reactants. Many data on the synthesis of mesoporous zeolites, their properties and catalytic applications were presented in the monograph of J. Garcia-Martinez and Kunhao Li [49]. Desilication of zeolite Y was much less frequently studied [35,50,51,52,53,54,55,56,57,58,59,60,61,62,63,64,65,66].

Most of the studies concerning hierarchical zeolites Y, including those that obtained them by incorporation of template micelles and by desilication, presented the results of structural and textural studies, as well as of catalytic tests. Even though most of the reactions catalyzed by hierarchical zeolites are catalyzed by acid sites, their acid properties of these zeolites were less frequently investigated. The present paper deals with the acidity of hierarchical zeolites Y, both these obtained by desilication and by template method. Assembly of zeolitic grains has also been briefly covered.

## 2. Experimental Methods Employed for the Acidity Studies of Zeolites

Many reactions in the industry are catalysed by acid sites, therefore characterization of acidity is a very important field of study in the chemistry of zeolites. The features of acidity that can be followed by various methods include concentration, acid strength, heterogeneity, and type of Brønsted and Lewis acid sites. The examination of acidity is performed using spectroscopic methods, such as infrared (IR) and nuclear magnetic resonance NMR spectroscopy, microcalorimetry, and thermoprogrammed desorption (TPD).

Solid-state NMR has been used for the characterization of acidity in zeolites and other materials for decades [67,68,69,70]. Different nuclei can be used for the characterization of SiOHAl groups, i.e., ^1^H, ^17^O, ^29^Si, ^27^Al, as well as other nuclei connected with probe molecules. ^1^H is the most prevalently used nuclei for direct observation of OH groups in MAS (magic angle spinning) NMR. In the ^1^H spectrum, signals from silanol groups and acidic Si-OH-Al groups can be easily discerned and provide quantitative information [71]. The content of Al in the framework of zeolites has a crucial impact on acidity, therefore ^27^Al and ^29^Si MAS NMR are frequently used. ^27^Al MAS NMR show the coordination of aluminum atoms in the sample and the chemical environment around Si atoms owing to the different content of Al atoms, which can be used to calculate of Si/Al in the framework [72]. In principle, the oxygen nucleus ^17^O could also be used to examine acidity, but the experiments with ^17^O are not prevalent due to low natural abundance and its high quadrupole moment [73]. The interactions of acid sites with probe molecules are also studied using various nuclei. ^1^H-NMR could be used to study interactions with different base molecules, for example pyridine, ammonia or acetonitrile, the latter of which show the adsorbate-induced shift of the signal from OH groups, which is the indication of the strength of acid sites. A growing interest is observed in using phosphorous-containing probe molecules like trimethylphosphine (TMP) or trimethylphosphine oxide TMPO [74,75]. ^13^C MAS NMR allows for the monitoring of changes in acetone when interacting with acidic OH groups [76,77,78]. 2D MAS NMR spectroscopy is also employed to study acidity, for example to probe Brønsted-Lewis site synergy [79], chemical exchange [80], or the spatial proximity between acid sites [81]. The details of NMR characterization of acid sites in zeolites are presented in recent reviews [82,83].

Temperature-programmed desorption (TPD) is prevalently used method to study acidic properties of catalytic materials [84,85]. It allows for the measuring of the amount of base molecules that can be desorbed from acid sites during thermal desorption, which indicates the concentration of acidic OH groups [84]. The most widely used probe molecule for these studies is ammonia, but other more reactive amines (ethylamine, n-propylamine, isopropylamine, tertbutylamine) have also been studied [86]. The biggest drawback of TPD is that it does not allow researchers to differentiate between Brønsted and Lewis acidity. The solution for this issue could be to use IR spectroscopy along with TPD [87,88]. TPD is also frequently used in tandem with micromalorimetry, TGA, and mass spectroscopy [89,90].

Microcalorimetry is another tool for measuring acidity, namely heats of adsorption of probe molecules. It has been widely used by Auroux et al. [85,91,92]. The measurement results in the plot of enthalpy of adsorption as the function of the coverage [93], from which the number of acid sites, their strength and heterogeneity can be deduced. But again, this method does not differentiate between Brønsted and Lewis acid sites, therefore the combination with other methods is used.

IR is by far the most widely used comprehensive method to study the acidity of zeolites. It offers easy discrimination between Brønsted and Lewis acid sites. The concentration of these acid sites can be determined if the extinction coefficients the diagnostic bands are known [94]. The information on the heterogeneity of OH groups deduced from the shape and splitting of Si-OH-Al band. The best method is to analyse the spectrum at low temperatures, possibly coupled with OH to OD exchange [95]. Pyridine and ammonia are the most important probe molecules for acidity studies. Pyridine is the most popular probe molecule, as it exhibits narrow diagnostic bands and is suitable for quantitative studies. It is also quite a bulky molecule, therefore it does not penetrate voids and channels with a diameter smaller than 0.5 nm For these materials, ammonia is used. However, ammonia is not so perfect molecule for quantitative studies, as N_2_H_7_^+^ dimers could be formed [96,97]. They are formed by the interaction of NH_4_^+^ ions with ammonia in the gas phase. However, the optimization of the experimental conditions makes quantitative IR studies of both Brønsted and Lewis acid sites feasible [96]. It has to be mentioned that quantitative studies in IR spectroscopy can be made only when the extinction coefficient is known [98]. Additionally, the catalytic behaviour can be predicted, if the reactant or similar molecules are sorbed and measured in IR experiment. Lercher [99] and Knozinger [100] presented the features that probe molecules should exhibit to provide a reliable examination of acidity. Low temperature IR experiments with CO and N_2_ give valuable insight into the acidity of the sample. CO interacts with the acidic OH group, and the shift of the corresponding OH band is observed. The shift is an indirect measurement of acid strength. Nitrogen is an interesting probe molecule because N≡N vibration is inactive in IR spectroscopy, but after interaction with acid sites, the narrow band appears in ca. 2330–2360 cm^−1^ [101]. The frequency of the band correlates with the acid strength of Brønsted and Lewis sites. Moreover, the $∆\nu_{\mathrm{OH}\cdot \cdot \cdot \mathrm{CO}}$ (100–150 cm^−1^) is smaller than those observed for CO, therefore, the shifted OH band may be observed also in the presence of coadsorbed other molecules like NH_3_ [101].

## 3. Desilication of Zeolites Y

Desilication is the most effective and economical method of production of hierarchical zeolites. As mentioned, most of the desilication research was done with zeolites of the MFI type. 

Desilication of zeolites Y is a more difficult task than other zeolites like MFI, BEA, and others. Pristine zeolite Y of Si/Al = ca. 2.5 has a high concentration of AlO_4_^–^ which protects the zeolite framework against OH^–^ attack. Therefore such a zeolite should be dealuminated before desilication. However, the structure of zeolite Y, which was dealuminted by steaming and acid leaching (of Si/Al above ca. 15), is so “injured” that it is sensitive to the reaction with OH^–^. Such zeolites were nearly destroyed even in diluted NaOH, the micropore volume diminished dramatically and an amorphous material of relatively good mesoporosity but very low acidity was obtained [50,51]. Similarly, even very diluted ammonia solution [59,66], which practically extracted neither Si nor Al, caused framework destruction, and material of reasonable mesoporosity was produced.

The addition of tetrapropylamonium or tetrabutylammonium ions (TPA^+^, or TBA^+^) to NaOH was a crucial modification of the desilication procedure. TPA^+^ or TBA^+^ that are known as pore-directing agents, protect the zeolite structure upon the mesopores formation, preserving the crystallinity and micropore system [35,51,53,62,63,64]. Zeolites Y desilicated with NaOH/TPAOH or Na/TBAOH mixtures showed only relatively small loss of crystallinity and microporosity, good mesoporosity, good acid properties, and good catalytic properties, which will be discussed in details in the next chapters. 

The reason why tetraalkylammonium cations stabilize zeolite structure is not well elucidated. Li and Shanz [102] revealed strong bonding of tetraalkylammonium ions with silica entities protecting zeolitic Si against an alkali attack. Therefore, TBAOH itself (Table 1) extracts only very small amounts of Si without a significant change of porosity. If tetraalkylammonium cations (known as a pore directing agent) are added to NaOH they act as an efficient pore-growth moderator during OH- assisted Si extraction [61]. It is possible that strong bonding of such cations with silica surfaces, shown by Li and Shanz [102], protects framework Si atoms against OH- attack.

If zeolite is treated with NaOH/TPAOH or NaOH/TBAOH mixtures TPA^+^ or TBA^+^ ions remain occluded inside zeolite cavities and they should be removed by burning out in air or oxygen above 720K. This problem does not exist in middle pore zeolites like MFI, the pores of which cannot host bulky TPA^+^ or TBA^+^ ions. Burning out TPA^+^ or TBA^+^ ions in zeolites Y causes further loss of crystallinity of zeolites and change of Al status.

### 3.1. Acid Properties of Dealuminated Zeolite Y (Parent Material for Desilication)

As mentioned, typical zeolite Y of Si/Al = ca. 2.5 is resistant to the treatment with bases being desilicating agents. This is due to the high concentration of AlO_4_^−^ tetrahedral, the negative charge of which repulses OH^-^. Therefore, zeolite Y should be dealuminated (until Si/Al reaches ca. 30) and subsequently desilicated in basic solutions. 

The zeolite which was the subject of desilication studies described in [51,52,53,54,66] was commercial CBV 760 (Zeolyst), and was steamed and acid-treated. The acid properties of this zeolite were subject of detailed studies [51]. The concentration of acid sites was determined by quantitative IR studies of pyridine sorption and are presented in Table 1. The concentrations of both Brønsted and Lewis sites were: 282 and 86 μmol/g respectively. The sum of the concentration of both kinds of acid sites is 368 μmol/g. This value was smaller than the amount of Al in zeolite of Si/Al = 31 (508 μmol/g) indicating, that some Al are engaged in the formation of either Brønsted or Lewis sites, but some may be inside clusters of extraframework Al species being inaccessible to probe molecules. Moreover, some AlO_4_^-^ tetrahedra may be neutralized by positively charged extraframework Al species.

Very important information concerns Si-O_1_H-Al groups. They were found to be very strongly acidic. The Δν of OH interacting by hydrogen bonding with CO was 354 cm^−1^. The acid strength of these hydroxyls was higher than of hydroxyls in HZSM-5 (Δν = 315 cm^−1^) [44] or HMOR (Δν = 310 cm^−1^) [32] and HBEA (Δν = 309 cm^−1^ – ref. [37]) which are known as strongly acidic zeolites. According to our best knowledge, only one zeolite, dealuminated mazzite, shows higher acid strength of Si-OH-Al (Δν = 378 cm^−1^ cm^−1^ ref. [38]). It may be supposed that this very high acidity may be related to the specific geometry of the environment of Si-O_1_H-Al bridge in the faujasite type structure. It should be noted that recent quantum-chemical density functional theory (DFT) simulations within an ab initio molecular dynamic (MD) approach applied to a periodic model of faujasite structure in actual experimental temperature evidenced also very high acidity of these hydroxyls. The calculated value $∆\nu_{\mathrm{OH}\cdot \cdot \cdot \mathrm{CO}}$ of 371 cm^−1^ agreed reasonably with experimental value (354 cm^−1^) [103].

**Figure 1:**
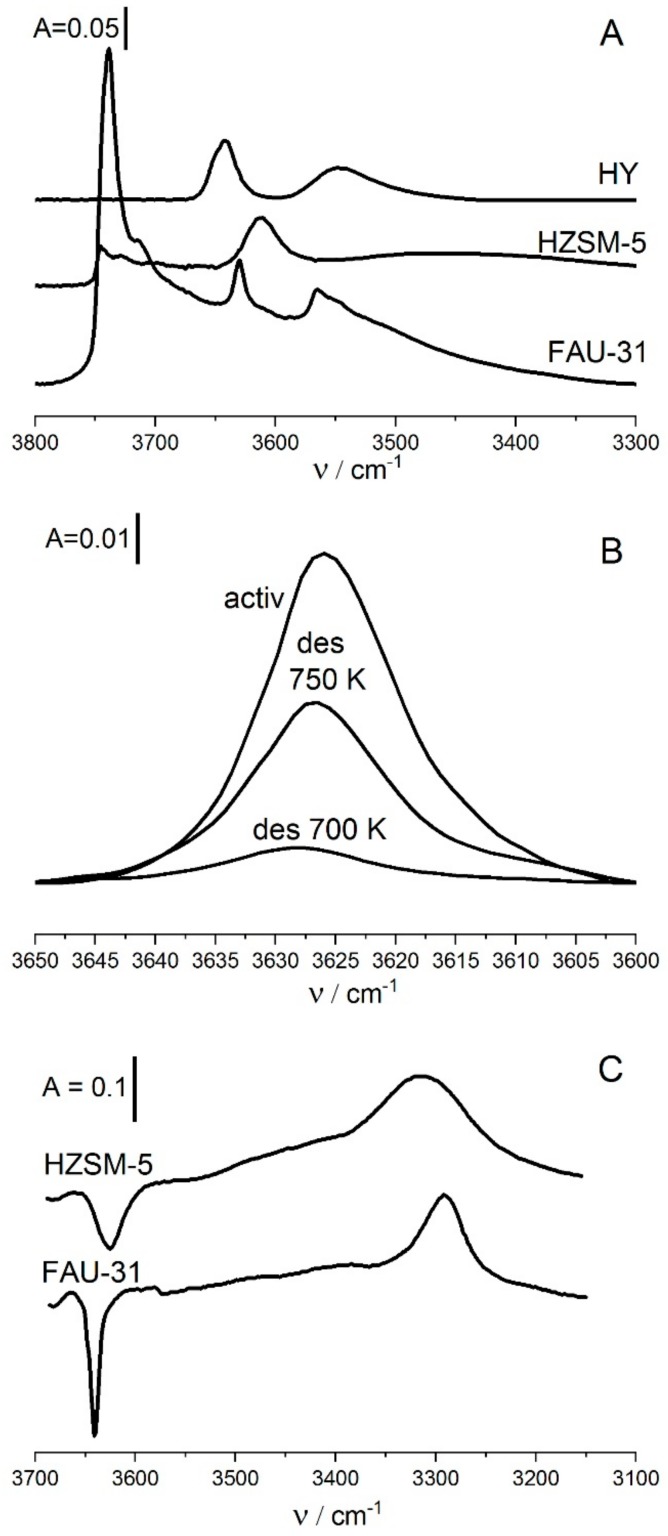
(**A**) The spectra of OH groups in zeolites HY (Si/Al = 2.8), HZSM-5 and FAU-31 (Si/Al = 31). Spectra are normalized to the same band intensity. (**B**) The spectra of 3620 cm^−1^ OH groups in FAU-31 recorded upon neutralization of all the hydroxyls by pyridine and desorption step-by-step at 700 and 750 K. (**C**) The spectra of 3620 cm^−1^ OH groups interacting by hydrogen bonding with CO at 170 K Figure adapted with the permission from [51].

Another very important property of Si-O_1_H-Al group is their homogeneity, all of them have the same acid strength. This statement is supported by three facts. The band of free Si-O_1_H-Al is more narrow than in typical zeolite HY (Si/Al = 2.5) and HZSM-5 (Si/Al = 30) (Figure 1A). The second argument is the fact, that the OH band which restores upon adsorption of pyridine and desorption step-by-step at increasing temperatures restores at the same frequency (Figure 1B). In the case of heterogeneous hydroxyls more weak hydroxyls of higher stretching frequency restore at lower temperatures than more acidic ones of lower acid strength. Therefore, the band of heterogeneous hydroxyls restoring at increasing temperatures shifts to lower frequency with the increase of desorption temperature. On the other hand, no such shift is observed for homogeneous hydroxyls. The fact, that for our FAU-31 no shift of restoring hydroxyls is observed (Figure 1B) indicates homogeneity of Si-O_1_H-Al. The third argument is a narrow band of OH interacting by hydrogen bonding with CO (Figure 1C), more narrow than for HZSZM-5. All these arguments evidence homogeneity of Si-O_1_H-Al in parent FAU-31 zeolite.

According to the results presented in [104], homogeneous hydroxyls are present in zeolites in which all of them have the same number of Al near the bridge and all have the same bridge geometry (the same bridge angle). According to the ^29^Si MAS NMR results, there are only Si(0Al) and Si(1Al) [54]. As Si(0Al) cannot form bridging hydroxyls, all the hydroxyls may be represented by the formula: (SiO)_3_Si-O_1_H-Al(OSi)_3_, all of them have only one bridging Al. All Si-O_1_H-Al have also the same bridge angle.

The dealuminated zeolite Y FAU-31 is the only zeolite we know, with very strongly acidic and homogeneous Si-OH-Al groups. Generally, in most of the zeolites, Si-OH-Al are heterogeneous (because there are hydroxyls of various number of Al near the bridge or of various bridge geometries), there are only a few zeolites with homogeneous OH groups: zeolites NaHA [105] and NaHX [106,107], these zeolites have homogeneous and very weakly acidic hydroxyls. Dealuminated zeolite Y is the only zeolite with homogeneous, but very strongly acidic hydroxyls.

### 3.2. Acid Sites in Desilicated Zeolite Y

As most of the catalytic reactions catalysed by hierarchical zeolites are catalysed by acid sites the characterization of acidity of zeolites is essential for catalytical applications. Most of the acidity studies were realized by TPD-NH_3_ method and IR studies of pyridine adsorption.

Li et al. [55] followed by IR spectroscopy the acidity of desilicated (by NaOH/TPAOH) zeolites Y which were first dealuminated by relatively mild steaming (at 600 °C) followed by citric acid treatment what caused the increase of SiO_2_/Al_2_O_3_ XRF from 5.2 to 12.1. Desilication decreased SiO_2_/Al_2_O_3_ XRF to 7.1, formed mesopores and caused the loss of ca. half of the protonic sites as well as the formation of an important amount of Lewis acid sites. The catalytic activity in triisopropylobenzene increased, indicating, that for this bulky molecule the improvement of transport inside mesopores is more important than the distinct loss of acid sites.

Less important loss of acid sites was observed [56] if zeolite Y, which was first dealuminated by steaming at mild conditions (550 °C) followed by the treatment of fluorosilicic acid/HCl mixture (Si/Al increased from 2.6 to 6.5), was next desilicated by NaOH treatment what caused the decrease of Si/Al to 3.4–4.6. Even though the Si/Al in both desilicated zeolites [55,56] were similar to the procedure applied in [56], this resulted in a less significant loss of protonic acidity.

On the contrary, Oruji et al. [57] reported a small increase of acidity (measured by TPD-NH_3_) in zeolite Y which was desilicated without previous dealumination. Desilication was done with NaOH without and with ultrasonication. While desilication with ultrasonication practically changed neither mesoporosity nor acidity, it preserved the microporosity (which was substantially decreased if zeolite was desilicated without ultrasonication). As a consequence, the cracking activity increased in ultrasonicated samples.

Very interesting results were obtained [58] if zeolite desilicated with NaOH was previously steamed and next treated with NH_4_F solution. NaOH treatment produced mesopores with only a small decrease of microporosity and caused an important increase of protonic acidity.

Rac et al. [64] reported microcalorimetric studies of ammonia sorption together with IR experiments of pyridine sorption. The effect of desilication on the acidity for ZSM-5, BEA, and USY zeolites was investigated. The authors discussed the distribution of the acid strength of sites obtained from microcalorimetry, and the concentration of acid sites from IR experiments. Desilication of zeolite ZSM-5 changed neither the concentration nor the acid strength of the acid sites. However, the desilication of zeolites BEA and USY decreased both concentration and acid strength of those sites. Desilication of zeolite USY in NaOH/TBABr mixture diminished only a little the micropore volume but produced mesopores of large volume. The catalytic activity of fructose dehydration increased.

**Figure 2:**
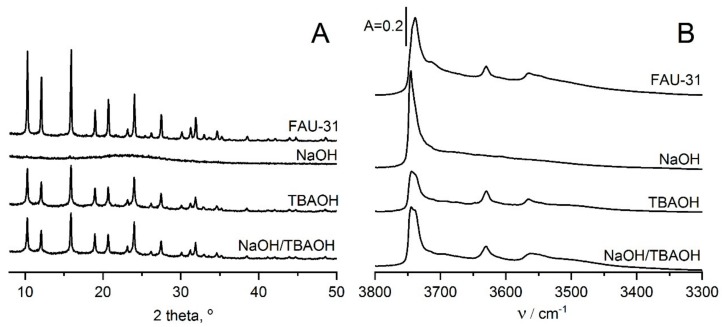
X-ray diffraction (XRD) diagrams (**A**) and the infrared (IR) spectra (**B**) of OH groups in zeolites FAU-31 parent and desilicated in NaOH, TBAOH and NaOH/TBAOH. Figure adapted with the permission from [51].

Detailed studies of zeolite Y of Si/Al = 31 (obtained by steaming followed by acid treatment) were realized by IR studies of pyridine and CO adsorption [51,53]. Zeolites were desilicated at room temperature with NaOH, TBAOH, and NaOH/TBAOH mixture. Even though all these bases show similar OH^-^ concentration, their reaction with the zeolite framework is different. X-ray diffraction (XRD) (Figure 2A) study evidenced, that NaOH caused complete amorphization of zeolite, on the other hand, the treatment with TBAOH and NaOH/TBAOH preserved crystallinity.

The spectra of hydroxyl groups in parent FAU-31, as well as in zeolites desilicated at room temperature with NaOH, TBAOH and NaOH/TBAOH mixture are presented in Figure 2B. The zeolites contain the 3730 cm^−1^ band of Si-OH, 3620 cm^−1^, and 3550 cm^−1^ bands of Si-O_1_H-Al and Si-O_3_H-Al acidic hydroxyls. The treatment with NaOH which destructed zeolite caused loss of both kinds of acidic hydroxyls, whereas both TBAOH and NaOH/TBAOH did not change OH spectra. 

The concentration of both Brønsted and Lewis acid sites was determined from quantitative IR experiments of pyridine adsorption. The acid strength was characterized by comparing the $∆\nu_{\mathrm{OH}\cdot \cdot \cdot \mathrm{CO}}$. The results of porosimetric and the acidity studies are compiled in Table 1. Desilication with NaOH which extracted 79% of Si, destructed microporosity and produced amorphous aluminosilicate of significant mesoporosity. It diminished the concentration of protonic sites and produced a large amount of Lewis sites. On the other hand, the treatment with TBAOH, which removed only a very small amount of Si and virtually remained with the porosity unaffected, (Table 1) changed the concentration of neither Brønsted nor Lewis sites as well. The treatment with NaOH/TBAOH mixture which extracted 43% of Si (Table 1) caused some decrease of microporosity and produced mesopores of significant volume. NaOH/TBAOH increased the concentration of the protonic sites (Table 1) from 282 to 310 μmol/g due to a decrease of the Si/Al. Rac et al. [64], who studied the desilication of zeolite Y by a NaOH/TPAOH mixture, reported a decrease of the concentration of protonic sites. It seems that the different acidity of our zeolites and those studied by Rac et al. [64] may be due to some differences in the desilication procedure and especially to the different pore-directing agents (PDA): TPAOH and TBAOH. The concentration of Lewis acid sites increased compared to the parent FAU-31.

The data on the acidity of desilicated zeolite Y can be compared with the results concerning the acidity of zeolites MOR [32], BEA [37], and MFI [44] desilicated with NaOH and NaOH/TBAOH. The treatment of these zeolites with NaOH caused the increase of protonic acidity due to the decrease of Si/Al. On the contrary, the treatment of dealuminated zeolite Y caused an important decrease of protonic acidity due to the destruction of zeolite framework (Figure 2). The desilication of zeolites BEA and MOR with NaOH/TBAOH mixture caused the increase of protonic acidity comparing to desilication using NaOH alone [32,37]. The situation is similar as in zeolite Y desilicated with NaOH/TBAOH. 

Very important information concerns the acid strength of the Si-O_1_H-Al groups. According to the data presented in Table 1, the $∆\nu_{\mathrm{OH}\cdot \cdot \cdot \mathrm{CO}}$ value for the parent FAU-31 zeolite is 354 cm^−1^. As mentioned above the acid strength of Si-O_1_H-Al groups is very high, only dealuminated mazzite contains more acidic hydroxyls. The treatment with TBAOH did not change the acid strength of Si-O_1_H-Al, but the most important result was obtained with zeolite desilicated with NaOH/TBAOH. The acid strength of Si-O_1_H-Al groups in these zeolites was practically the same as in parent FAU-31. Our earlier studies of desilication of zeolites ZSM-5 [44], BEA [37] and MOR [32] evidenced, that the acid strength of Si-OH-Al decreased upon desilication. It was explained by the heterogeneity of the OH groups in those zeolites and the presence of more and less acidic hydroxyls. It was also evidenced that desilication removed small amounts of Al atoms, and the Al atoms removed in the first order were those responsible for formation the most acidic Si-OH-Al. In our parent FAU-31 (which was dealuminated before the desilication) all the Si-OH-Al were homogeneous and the alkali treatment, which extracted some small amounts of Al (see Table 1), changed only the number but not the average acid strength of OH.

The formation of mesopores in zeolite Y with some increase of concentration of protonic sites, the acid strength of which is still very high upon desilication, caused a significant increase of conversion in α-pinene isomerization [51]. While zeolite treated with NaOH showed lower activity even though mesoporosity increased, the zeolite desilicated in NaOH/TBAOH mixture showed distinctly higher conversion than the parent, non-desilicated zeolite. The α-pinene isomerization produces camphene and limonene (which undergoes further reactions) as well as unidentified products (including oligomeric deposits) [51]. The contribution of camphene and limonene reaction paths depends mostly on the acidity. The zeolites of comparable and very high acidity, but different porosity (parent FAU-31, and zeolites treated with TBAOH and NaOH/TBAOH) show a comparable contribution of camphene and limonene (with further products), whereas for the zeolite desilicated with NaOH of lower acidity, the contribution of camphene was higher and the selectivity to limonene (with products of further reactions) was lower.

The results concerning acidity and catalytic activity of desilicated zeolites Y presented above were obtained for zeolite Y of Si/Al = 31 which was treated at room temperature with NaOH/TBAOH containing 10 mol % of TBAOH. The study was undertaken to optimize the condition of desilication and producing zeolite of optimal porosity, acidity and catalytic activity [53]. The NaOH/TBAOH mixtures of various proportions between both bases were used and desilication was realized at various temperatures (in the range RT – 373 K). The results are presented in Table 2 and Table 3. According to the data presented in Table 2 and Table 3, the optimal conditions for desilication i.e., conditions in which zeolite of optimal microporosity, optimal mesoporosity, acidity and optimal catalytic properties in α-pinene isomerization are: treatment at 353K of zeolite of Si/Al = 31 with NaOH/TBAOH containing 10 mol % of TBAOH. It was also taken into account that TBAOH is a rather expensive chemical, so rather low contents of this base are preferred.

**Figure 3:**
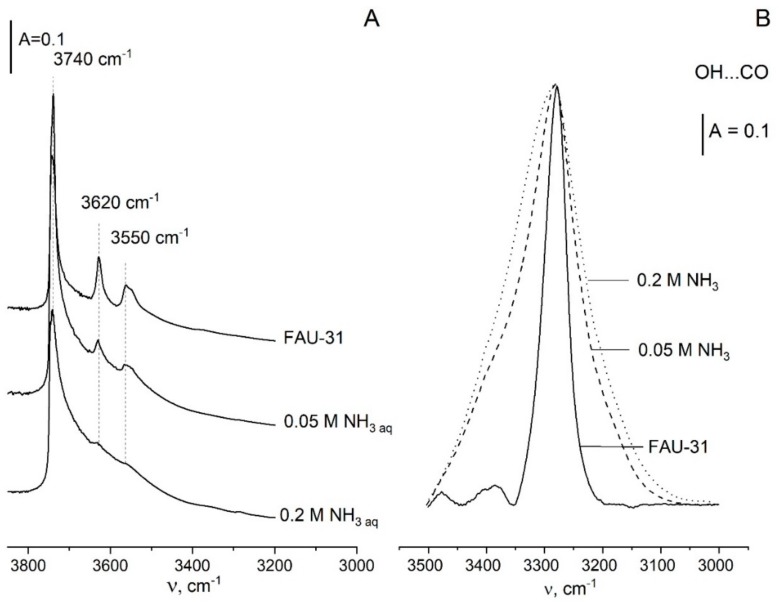
IR spectra of free OH groups (**A**) and OH groups hydrogen bonded with CO (**B**) in the parent zeolite and samples treated by ammonia solutions. Figure adapted with the permission from [66].

Y zeolite of Si/Al = 31 was also treated with 0.05 and 0.2 M ammonia solutions [66]. Ammonia extracted only very small amounts of Si but caused significant destruction of the zeolite framework. Microporosity of zeolite was distinctly reduced and some mesopores were formed. The IR bands of zeolitic Si-OH-Al groups diminished distinctly (Figure 3A) as well as the concentration of protonic sites determined in IR studies of pyridine adsorption (Table 4). On the other hand, Lewis acidity increased. The acid strength of remaining Si-OH-Al groups decreased slightly, and these OH groups became heterogeneous (as evidenced by broadening of IR band of OH groups interacting with CO). Zeolite treated with ammonia solution showed an increase of catalytic activity in α-pinene isomerization if compared with parent zeolite, even though acidity decreased. This may be related to the increase of mesoporosity. Such zeolitic materials might therefore constitute promising catalysts for the liquid phase reactions, where the presence of an additional mesopore system is desirable. Finally, from an economic standpoint, the treatment of zeolites with inexpensive solutions of ammonia seems to be much more convenient than a synthesis route of hierarchical zeolites based on the costly tetrabutylammonium hydroxide.

### 3.3. Hydroxyl Groups of Extremely High Acidity in Desilicated Zeolite Y

**Figure 4:**
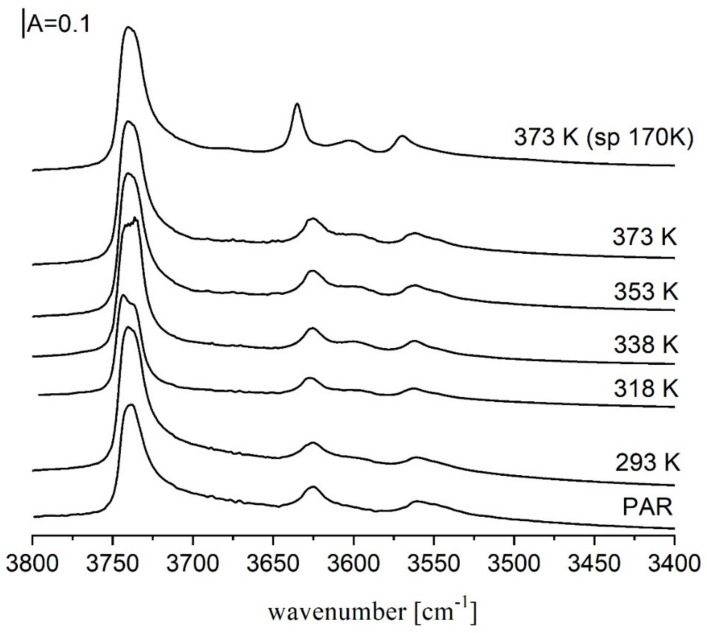
IR spectra of OH groups in zeolite Y desilicated in NaOH/TBAOH at various temperatures. All the spectra were recorded at room temperature, only the top spectrum was recorded at 170 K. Figure adapted with the permission from [53].

**Figure 5:**
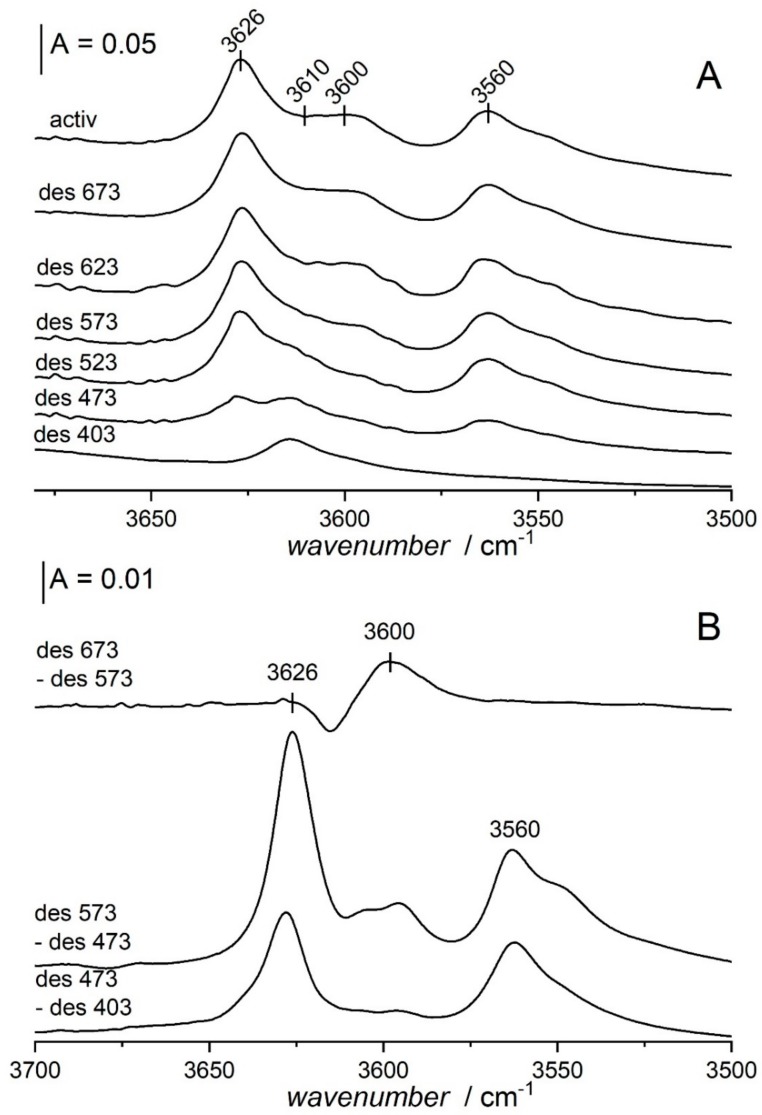
(**A**) The spectrum of zeolite Y desilicated and calcined in atmospheric air recorded upon the activation (top spectrum) and the spectra recorded upon the sorption of NH_3_ followed by the desorption at: 403, 473, 523, 573, 623 and 673 K (from bottom to top). (**B**) The difference spectra: differences between two desorption steps. Figure adapted with the permission from [52].

The spectra of OH groups in zeolites Y desilicated in NaOH/TBAOH at temperatures above 318 K and next calcined at 790 K show not only 3550, and 3620 cm^−1^ bands of acidic Si-O_3_H-Al and Si-O_1_H-Al hydroxyls respectively, but also a new band at ca. 3600 cm^−1^ [52,53]. This band is the best seen in the spectrum recorded at 170 K. (Figure 4). This band represents strongly acidic hydroxyls what was evidenced in the experiments of ammonia desorption (Figure 5). 3600 cm^−1^ hydroxyls restore at highest temperatures of ammonia desorption, which proves that they are more acidic than those represented by 3550 and 3620 cm^−1^ bands.

**Figure 6:**
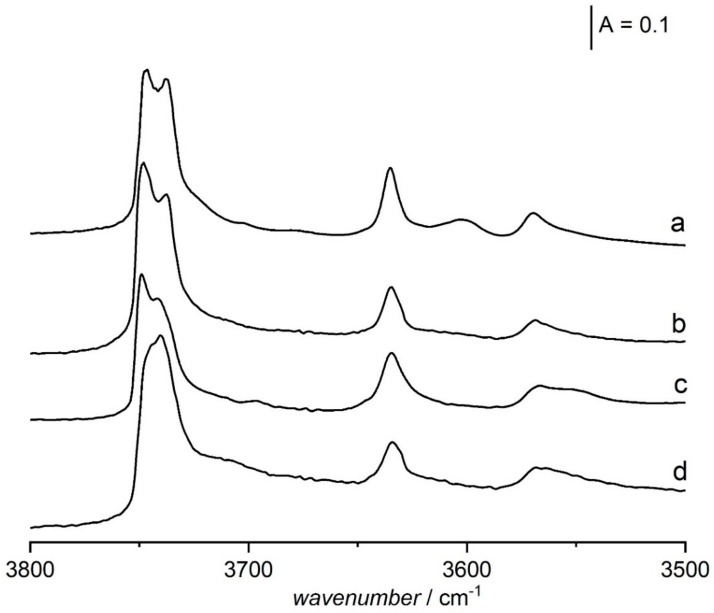
The spectra of OH groups in zeolite Y desilicated in which TBA^+^ ions were removed by: a - calcination in atmospheric air at 790 K, b - oxidation in oxygen at 790 K, c- calcination in vacuum at 720 K, d - calcination in dry air at 790 K. All the spectra were recorded at 170 K. Figure adapted with the permission from [52].

OH groups of very high acid strength (vibrating at 3600 cm^−1^) are also present in zeolite USY produced by steaming. It was postulated by many authors, e.g., [108], that these hydroxyls were formed by the reaction of water with the zeolitic framework what caused the extraction of some Al. Such extraframework Al interacted with Si-OH-Al what resulted in the significant increase of their acid strength. It may be supposed that a similar mechanism increases the acidity of Si-OH-Al in desilicated zeolite Y. This zeolite was desilicated in NaOH/TBAOH, Na^+^ was next exchanged to NH_4_^+^ and, finally, NH_4_^+^ ions were decomposed and TBA^+^ ions were removed by calcination in atmospheric air at 790 K. It may be supposed, that 3600 cm^−1^ OH groups were produced during calcination, even though water was not supplied during the calcination procedure. IR studies [52] evidenced that water, which extracts Al from the zeolite framework during calcination, was present in atmospheric air. It was shown [52] that, if oxidation of TBA^+^ ions was done by calcination in oxygen or in dry air the 3600 cm^−1^, hydroxyls were not formed (Figure 6). This extraframework Al interacting with Si-OH-Al groups increases their acid strength according to the mechanism proposed by Makarova et al. [108] for zeolites USY. Normally, the treatment of pristine zeolite Y (non-desilicated) in the flow of atmospheric air at high temperatures does not cause Al extraction. Similarly, in the zeolite Y desilicated with NaOH/TBAOH at room temperature, calcination in atmospheric air did not produce 3600 cm^−1^ hydroxyls. It seems possible that the formation of very strongly acidic 3600 cm^−1^ OH groups takes place during the treatment of water present in atmospheric air with the zeolitic framework which was already destabilized by the treatment of NaOH/TBAOH at temperatures higher than room temperature.

**Figure 7:**
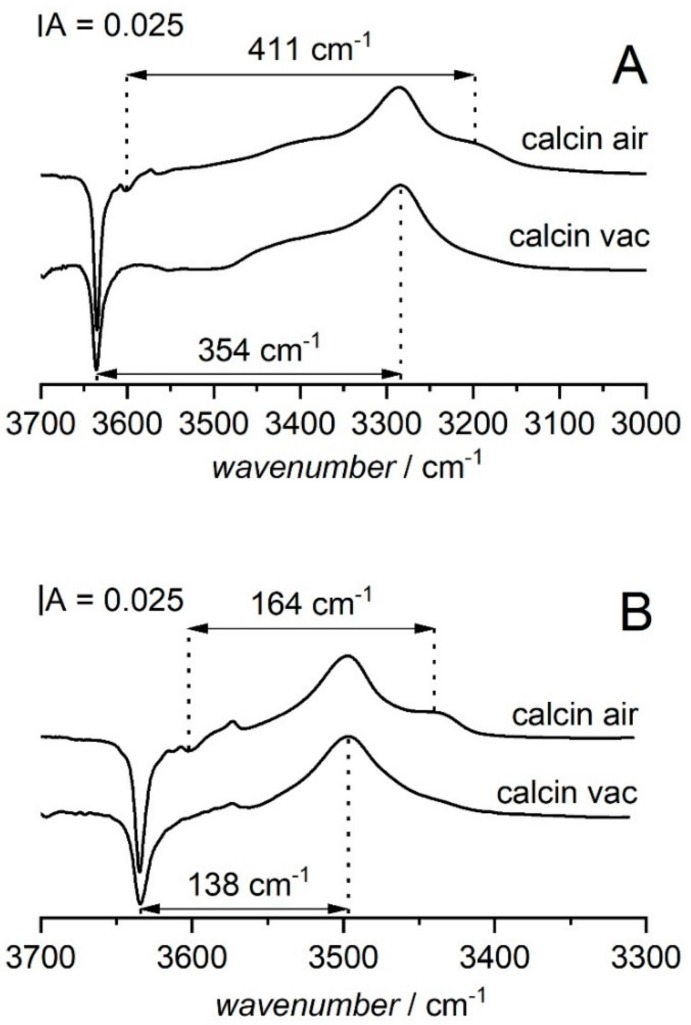
(**A**) The difference spectra of OH groups in zeolite calcined in air and in vacuum interacting with CO. (**B**) The difference spectra of OH groups in zeolite calcined in atmospheric air and in vacuum interacting with N_2_. All the spectra were recorded at 170 K. Figure adapted with the permission from [52].

Very important information on the acid strength of hydroxyl groups was obtained in the IR experiments in which the hydrogen bonding of OH groups with CO and N_2_ molecules was investigated [52]. Two zeolites Y desilicated in NaOH/TBAOH at 373 K were studied. One zeolite was calcined in vacuum at 790 K and the second one was calcined in atmospheric air at the same temperature. The first zeolite did not contain 3600 cm^−1^ groups and the second one contained these hydroxyls. The results obtained with CO sorption are presented in Figure 7A. The difference spectra (spectra after CO sorption minus spectra before sorption) of both zeolites show a maximum of Si-O_1_H-Al groups interacting with CO for which the frequency shift $∆\nu_{\mathrm{OH}\cdot \cdot \cdot \mathrm{CO}}\;$= 354 cm^−1^ is the same as for the parent zeolite before desilication, indicating that the acid strength of Si-O_1_H-Al groups which was very high in zeolite before desilication did not change upon desilication. However, the most important observation was the submaximum at ca. 3200 cm^−1^ which is the band of 3600 cm^−1^ hydroxyls interacting by hydrogen bonding with CO. The most striking fact is the very big value of frequency shift $∆\nu_{\mathrm{OH}\cdot \cdot \cdot \mathrm{CO}}\;$= 411 cm^−1^ indicating very high acid strength of these hydroxyls. According to ref. [50] the $∆\nu_{\mathrm{OH}\cdot \cdot \cdot \mathrm{CO}}$ value is the highest in all the chemistry of zeolites.

The information on the acid strength of OH groups was also obtained by following their hydrogen bonding with N_2_ [52]. The difference spectra of OH groups in desilicated zeolites Y calcined in a vacuum and in the air interacting with N_2_ are presented in Figure 7 B. For the zeolite calcined in vacuum the 3626 cm^−1^ Si-O_1_H-Al band shifts by 138 cm^−1^, whereas for the zeolite calcined in the air the 3600 cm^−1^ band shifts by 164 cm^−1^. This value is significantly higher than in all other zeolites including USY and dealuminated mazzite $∆\nu_{\mathrm{OH}\cdot \cdot \cdot N_{2}}\;$= 143 and 144 cm^−1^ respectively), which are known as very strongly acidic.

The information on the electroacceptor properties of the adsorption site may be also obtained by comparing the values of the stretching frequency of C≡O molecules. Generally, the more electroacceptor the adsorption site is, the higher the C≡O frequency is [99]. The frequencies of CO band in molecules interacting with acidic Si-OH-Al groups in zeolites HZSM-5, HY dealuminated, HY desilicated, as well as in zeolite USY and dealuminated mazzite are presented in Table 5. The CO frequency in molecules interacting with 3600 cm^−1^ in desilicated Y (2183 cm^−1^) is higher than in all other zeolites including very strongly acidic USY and dealuminated mazzite. This agrees well with very high values of $∆\nu_{\mathrm{OH}\cdot \cdot \cdot \mathrm{CO}}$ and $∆\nu_{\mathrm{OH}\cdot \cdot \cdot N_{2}}$ frequency shifts.

By summing up one can state that the zeolite Y dealuminated (Si/Al = 31) which was the parent material for desilication contains very strongly acidic (more acidic than in HZSM-5) and homogeneous Si-O_1_H-Al groups. Desilication in the mixture of NaOH/TBAOH above 318 K, which extracts about half of the Si and produces mesopores of large volume, produces also a new kind of hydroxyls (3600 cm^−1^ ones) of extremely high acidity - more acidic than in all the zeolites known. We suppose, that the 3600 cm^−1^ hydroxyls were formed by the reaction of atmospheric water with the framework of zeolite at high temperature during calcination. Such a reaction is (in our opinion) possible only upon some destabilization of the framework by the desilication process carried out at elevated temperatures (above 318 K). It is interesting to say that the reaction of water with pristine (not dealuminated nor not desilicated) zeolite Y of Si/Al ca. 2.5 also takes place at high temperature, but in this case, large amounts of water must be supplied during the steaming procedure, whereas, in our desilicated zeolite, even the small amount of water present in atmospheric air is sufficient to extract some Al.

### 3.4. Status of Al and origin of Lewis Acid Sites in Desilicated Zeolites

We studied the status of Al in zeolites desilicated with NaOH and NaOH/TBAOH. We followed also how the status of Al changed upon calcination that decomposed NH_4_^+^ ions, created protonic sites, and, at higher temperatures, caused dehydroxylation. The experimental methods were ^27^Al MAS NMR spectroscopy as well as by IR spectroscopy with NH_3_ and CO as probe molecules [54]. Desilication removes both Si and Al from zeolite, but while extracted Si species are prone to self-aggregation and remain in solution, Al species prefer to be adsorbed on zeolite surface. NMR studies evidenced that the contribution of bulk tetrahedral zeolitic Al decreases upon desilication and the contribution of modified tetrahedral nonzeolitic Al forms (similar as in amorphous aluminosilicates) increases. This is important since the effective enrichment of the concentration of surface Al forms is frequently claimed to be responsible for hindering excessive mesopore formation. Quantitative IR study evidenced that Al extracted from zeolite is next reinserted back as tetrahedral nonzeolitic Al and shows ion exchange capacity. This statement was supported by the fact that the amount of NH_4_^+^ ions introduced by ion exchange was found to be the same as the amount of Al. NH_4_^+^ ions introduced at the ion exchange process decompose during the calcination forming protonic sites. Some of these protonic sites dehydroxylate, forming Lewis acid sites according to the stoichiometry typical of zeolites (two protonic sites condensate producing one Lewis site and water molecule). Generally, in “typical” non-desilicated zeolites acidic hydroxyls are resistant at temperatures below 750 K (only small amounts of Si-OH-Al is lost); however, hydroxyls related to nonzeolitic tetrahedral Al, which was reinserted to zeolites, are much more prone to dehydroxylation.

## 4. Surfactant Templating of Zeolite Y

Surfactant molecules can be added to the synthetic gel before the hydrothermal treatment or used to modify existing zeolite crystals. In the first approach, phase separation is often observed, resulting in partly-amorphous and partly-crystalline heterogeneous material. This issue may be solved by using silylated surfactants that covalently bind to growing zeolite crystals and compromise two functions: structure direction and mesopore formation at the same time. The drawback of this method is the high cost of organic molecules and often-observed aggregation of nanosized zeolites [23]. The second method is more versatile. The idea of this approach is to modify the zeolite by the surfactant treatment in alkaline solutions. It has proven to be an effective way to control the shape and size of pores. The porosity can be tuned by a careful choice of surfactant and optimization of the conditions of the treatment. These materials compromise high concentration and strength of acid sites with additional mesopores, which facilitate the accessibility of acid sites to reactant molecules. The pores generated using this method are uniform in shape. Zeolites Y, L, ZSM-5, and Beta (among others) have been treated this way. Using surfactants for pore generation has been reviewed in [23]. Here, we report recent papers focusing on zeolite Y.

### 4.1. Surfactant Templating during the Synthesis of Zeolite Y

Surfactants have been used as the ingredient to synthesize mesoporous zeolite Y catalysts used for catalytic cracking. Tempelman et al. [19] used organosilane dimethyl octadecyl-(3-trimethoxysilylpropyl)-ammonium chloride (TPOAC) to prepare mesoporous zeolite Y. It has been compared with its microporous counterpart. They used ^27^Al MAS NMR, which showed the presence of tetrahedral and octahedral Al in both samples. ^1^H MAS NMR was engaged to differentiate a few types of OH groups and calculate their concentrations. Mesoporous zeolite turned out to have a lower number of Brønsted sites, AlOH and SiOH groups than the microporous zeolite. The acidity was examined as well using FT-IR spectroscopy with adsorption of deuterated benzene. The parent materials showed characteristic bands for OH groups in supercages (3631 cm^−1^) and sodalite cages (3550 cm^−1^). After the adsorption of C_6_D_6_, the intensities of the OH bands decreased, and new OD +bands at 2630 cm^−1^ and 2680 cm^−1^ develop. ^1^H-NMR and IR showed that mesoporous zeolites have fewer protons located in sodalite cages than in supercages, which was contrary to microporous zeolite Y. The concentration of OH groups was calculated by deconvolution of OD stretching bands. The values were lower than those calculated from Si/Al from ^29^Si MAS NMR. It was ascribed to post-treatments of the materials and by the influence of extra framework aluminum (EFAL) species, which could partially compensate the negative framework charge. Generally, the mesoporous zeolite was found to be less acidic than its microporous counterpart. Microporous and mesoporous zeolites Y has been used to prepare catalysts by mixing them with kaolin (filler) and alumina sol (binder). The acidity of the non-modified mixture measured by IR and deuterated benzene was directly linked to the content of the zeolitic phase in the mixture. The mixed samples were steamed and calcined to simulate the deactivation in the fluid catalytic cracking (FCC) regenerator. These samples exhibited no Brønsted acidity by H/D exchange but generated some pyridinium ions when interacting with pyridine. The mixture with microporous zeolite exhibited more protonic sites than mesoporous zeolite-containing one, but in general, their content was low. These protonic sites have been ascribed to amorphous silica-alumina that were generated by steaming and regeneration. The temperature for a conversion of 40% in n-heptane isomerization was found to be around 600K for both samples, which is typical for amorphous Si-Al. In FCC tests, the samples with microporous zeolite exhibited however higher conversion, whereas for the sample with mesoporous zeolite diesel yield was higher.

Zhao et al. [20] used nonionic surfactant, Pluronic P123, as the additive to a standard aluminosilicate gel and hydrothermal treatment in order to obtain mesoporous zeolite Y. The samples have been ultra-stabilized by steaming and mixed with kaolin and alumina gel, for use in catalytic cracking. The authors compared the features of the standard ultra-stable zeolite Y (US-Y) and samples with additional mesoporosity generated by P123. NH_3_-TPD and pyridine IR results of composite catalysts showed that the acid strength and concentrations are higher when a standard USY zeolite was used to prepare the catalyst. The authors claimed that the acidity was not crucial for cracking, but high porosity was. The sample with the highest mesoporosity, and therefore the best diffusion properties, exhibited the highest conversion and lowest coke yield.

### 4.2. Post-Synthetic Surfactant Templating of Zeolite Y

Garcia-Martinez [25] developed an efficient method to introduce mesoporosity to zeolites Y using cetyltrimethylammonium bromide (CTAB) treatment in alkali solution. Organic template forms micelles that, after burning, form narrow and uniform mesopores. The method has been improved by the treatment with citric acid which caused only minor dealumination and prevented from the destruction of the zeolite framework, allowing at the same time for opening the Si-O-Al bonds and creating mesopores in zeolites with Si/Al ca. 2.5. Aluminum atoms in mesoporous zeolite Y obtained by the surfactant-templating method occupy only tetrahedral positions. The acidity of mesoporous zeolites Y was generally preserved, in reference to microporous material. The acidity of steamed mesoporous zeolite Y observed via NH_3_ TPD was similar to commercial USY. Both materials exhibited also good hydrothermal stability and high performance in FCC reactions.

Sachse et al. [29] provided a deep examination of the mechanism of mesopore formation in surfactant templating method. They treated ultra-stable zeolite Y (Si/Al = 35) with CTAB and other surfactants with bigger surfactant heads in basic media. The basicity of the medium in CTAB mixture was a crucial factor determining the mesopore formation, as it created SiO^-^ charges in the framework that attracted positively charged cations in surfactant molecules. The molecules need to have the ability to transport through the microporous channels, therefore surfactants with big heads did not form mesopores. When surfactant molecules were situated inside the microporous structure, the zeolite framework reorganized by the short-scale breaking of the bonds and reconstruction of them around the micelles. The materials have been characterized by transmission electron microscopy (TEM), N_2_ and Ar BET sorption, which proved that mesoporosity was produced without deterioration of the crystal structure. The reflexes in XRD pattern showed lower intensity in comparison with parent sample, but the authors ascribed it to the presence of mesopores, which decreased the repetition of the unit cell. No amorphous material or material loss is observed. The acidity of the samples has been examined by pyridine adsorption and IR spectroscopy. Mesoporous samples showed a slight decrease in Brønsted acidity, connected with some decrease in microporosity and crystallinity, although even the sample that possesses the highest volume of mesopores, retained 70% of the proton acidity of the parent sample. They also showed that mesoporosity in the starting material was not essential for successful mesopore formation using surfactant-templating, because this treatment was also feasible in a standard NaY zeolite.

Silva et al. [27] used CTAB molecules to generate mesoporosity in USY zeolite with Si/Al ratio of 15. They concluded that CTA^+^ cations acted similarly to TBA^+^ cations in desilication, i.e., protected zeolitic structure from deterioration by too intense dissolution of zeolite framework by OH^-^ ions. However, hydroxyl ions were essential for mesoporosity to be created, therefore a simultaneous use of CTA^+^ and OH^-^ was needed for obtaining crystalline and mesoporous material. The obtained material exhibited a lower concentration of acid sites compared to the parent USY sample calculated from NH_3_ TPD experiments. Si/Al ratio of materials did not change, indicating that desilicated Si was associated with micelles, and recrystallized. The authors also noticed three types of aluminum visible in ^27^Al MAS NMR, i.e., standard tetrahedral Al, distorted tetrahedral Al, and octahedral Al. The samples have not been examined in catalytic experiments.

The interplay between desilication by OH^-^ ions and mesopore formation by CTA^+^ has been scrutinized by Mehlhorn et al. [28]. Their starting material was dealuminated zeolite Y with Si/Al = 15. The amount of NaOH in reference to Si during the surfactant treatment was the parameter examined. Porosity studies included N_2_ and Ar sorption BET. The mesopore formation began at NaOH/Si = 0.0625. The volume of mesopores increased with NaOH/Si, but the volume of micropores decreased at the same time. For 0.0625 < NaOH/Si < 0.10, it was possible that crystalline walls were present in the material. For higher values of NaOH/Si those materials were “a mosaic of mesostructured amorphous domains and zeolitic crystalline nanodomains distributed over the whole crystal”. XRD diffraction peaks were not present for materials NaOH/Si > 0.175. Acidity was measured by NH_3_ TPD. Only the peak at 380 °C has been taken into account. The concentration of acid sites decreases with an increase of OH- ions, and therefore, increased with the zeolite content. The samples have not been examined in catalytic experiments.

Tan et al. [21] used a mixture of nanoclusters of zeolite Y, kaolin and CTAB molecules to produce composite materials with micro-/meso-/macroporosity. Acidity was examined via NH_3_ TPD and IR spectroscopy. Concentration and acid strength in a composite was lower than in a typical zeolite Y. The authors proved that in crude oil cracking reaction, the porosity was more important than acidity, as highly-porous zeolite Y exhibited higher conversion than its standard counterpart.

Hierarchical zeolites of faujasite type were also used as basic catalysts [26]. Zeolites X and Y were first treated with citric acid which created defects in the framework that facilitated braking Si-O-Al bonds during CTAB treatment in alkali solution [29]. The acid step was essential for crystallinity and can control the degree of mesoporosity. The authors showed that the higher concentration of citric acid was used, the crystallinity of the formed mesoporous sample was lower, and the number of defects was higher. After ion exchange with K^+^ and Cs^+^ ions, the basicity of the materials has been examined by using IR spectroscopy. Adsorption of pyrrole and acetylene and observation of the shift of N-H and C-H stretching vibration bands, respectively, showed that the basicity of Cs, K form was higher than Na form, and was generally the function of Al content in the sample. The samples of mesoporous zeolites Y and X have been examined in the transesterification of rapeseed oil with methanol. The main conclusion of the article was that the mesoporosity was not a defining factor for this reaction. Mesoporous zeolite Y contained more mesopores, whereas zeolite X was more efficient. It was due to a lower Si/Al ratio, therefore higher ion-exchange capacity and higher basicity of the framework.

## 5. Hierarchical Zeolites Y Obtained by Assembly of Zeolitic Grains

Another strategy of production of hierarchical zeolites Y is an assembly of small zeolitic grains. This method is less expensive than the synthesis with templates and produces zeolites of good catalytic properties in reactions of bulky molecules.

Travkina et al. [10] proposed to generate macro- and mesoporous zeolite Y by modification of granules that contain a mixture of microporous zeolite with kaolin. After calcination, the granules were crystallized in sodium silicate solution, ion-exchanged for NH_4_^+^, dried, and calcined in dry air. No matter what was the primary zeolite content (30% or 60%), the final crystallinity of the samples was very high, ca. 95%. According to the authors, mesopores were present due to nanocrystals of zeolites, and macropores were formed as a result of larger crystals clustering. Meso- macroporous samples exhibited lower acidity measured in NH_3_ TPD experiments in comparison to standard protonic zeolite Y (HY), which was ascribed to clustered crystals that make some acid sites unavailable to ammonia molecules. IR spectroscopy for mesoporous zeolite Y showed composed bands of acidic hydroxyls and OH groups bound to the EFAL species. The spectrum was similar to zeolite Y produced by steaming. IR studies of CO adsorption showed that mesoporous zeolite had slightly higher acid strength of OH groups in supercages than standard HY but lower than in dealuminated Y. Adsorption of CO provided also information on the Lewis acid sites and their acid strength. The concentration of medium and weak L.a.c. was found to be three times higher than in microporous material. Nevertheless, mesoporous zeolite was superior catalysts for the multicomponent reaction of propanol, formaldehyde, and ammonia producing lutidine.

The strategy of formation of mesoporous zeolites without organic templates has been introduced by Du et al. [11] The authors present a method of synthesis of a flower-like structure of hierarchical zeolite Y, only by using a hydrothermal treatment. By careful optimization of the composition, they were able to obtain primary nanocrystals aggregates that formed a flowerlike morphology. Intercrystalline voids composed mesopores and macropores of broad pore size distribution from 10 to > 100 nm. ^27^Al MAS NMR spectra showed only tetrahedrally coordinated atoms, although signals from distorted tetrahedral Al were also visible. Ammonia TPD showed that the number of acid sites, as well as their strength, depended on the content of the zeolitic phase in the materials, as most of the acid centers were located in microporous channels. Si/Al ratio in the gel changed the morphology of the crystals and did not affect the amounts of the acid sites. Catalytic cracking of isopropylbenzene showed a strong dependence of the conversion with the external surface area.

## 6. Conclusions

From the point of view of catalytic applications, a material with ideal acidic properties should be found at the crossing point of the zeolitic route ensuring high concentration and strength of acid sites, and the mesoporous route guaranteeing the high accessibility of acid sites to reagent molecules. Generally, several main routes leading to the formation of mesopores were applied. One of these routes is the use of surfactants in the synthetic procedure (“bottom up”), another method uses surfactants during a post-synthetic treatment (“mesostructuring”). The third route utilizes a “top down” strategy. Most of the studies realized in the “top down” route use desilication which produces mesopores of various dimensions and changes the composition of zeolite. Desilication decreases Si/Al, causes the extraction of both Si and Al, but while Si remains in the solution, Al tends to be reinserted to zeolite grains. As a consequence, the acid properties of zeolite change. NaOH, which extracts ca. 80% of Si causes complete amorphization, loss of microporosity, and loss of protonic acidity. Mesoporous, amorphous material was obtained. Even though mesopore volume was relatively big, catalytical properties of zeolite treated with NaOH were poor due to the loss of most of the protonic sites. On the other hand, treatment with ammonia solution which produces also mesopores diminishes the concentration of protonic sites, but their acid strength is only a little reduced. The catalytic activity in α-pinene increases if comparing with parent zeolite, indicating, that for the reaction of bulky molecules the accessibility of sites is more important than acidity.

The best results were obtained if desilication of zeolites Y is realized with NaOH/TBAOH mixture. The optimal composition and optimal desilication temperature were elaborated, and finally, zeolite of very good mesoporosity, with its concentration of protonic sites higher than in parent zeolite, was obtained. Most of the mesoporosity and high acid strength of Si-OH-Al was preserved. Therefore the catalytic activity in α-pinene isomerization was significantly higher than in parent material.

A very important observation was the formation of Si-OH-Al of extremely high acidity vibrating at 3600 cm^−1^. Their acid strength ($∆\nu_{\mathrm{OH}\cdot \cdot \cdot \mathrm{CO}}\;$= 411 cm^−1^) was the highest in all the chemistry of zeolites. They were formed during the burning off TBA^+^ ions in the air by the reaction of atmospheric water with the framework. The process similar to the “steaming” of zeolite causes the extraction of the same Al from the framework. Such extraframework Al increases the acid strength of hydroxyls, as it happens in the case of USY zeolites.

Surfactant-assisted mesopore formation allows for homogenously sized mesopores. Surfactants can be added before the hydrothermal synthesis or in the form of post-treatment. The first method uses various organic templates, like organosilanes and non-ionic surfactants (among others) and results in the material of considerably lower Brønsted acidity than zeolite Y. The latter method is widely used with CTAB molecules. These molecules are perfect because they are small enough to penetrate the microporous structure. Inside, they form micelles that, after burning out, will form mesopores. However, for them to do so, the basic environment is essential. Under these conditions, SiOHAl bonds can be partially broken, which enables the formation of mesopores around micelles. Standard zeolite Y has low Si/Al (Si/Al = ca. 2.5), therefore the interaction with OH- ions is weak because hydroxyl ions are repelled from the structure due to high negative charge of the framework. It hinders the mesopore formation. This issue has been tackled by the pretreatment of the zeolite with acid. Therefore, both standard zeolite and dealuminated zeolite Y could be used to obtain mesoporous material. CTAB molecules also protect the zeolitic structure from the damage done by the excess of OH^-^ ions. In fact, a too high amount of hydroxyl ions during the treatment with a surfactant could produce amorphous material. The acidity of the obtained material is connected with its crystallinity. When the conditions of the treatment are carefully optimized, crystallinity is preserved, and only a slight decrease in Brønsted acidity is observed. Although in many reactions, mesoporosity is more important than acidity. Mesoporous zeolites are great hosts for alkali metal ions and proven effective in the production of biofuel. In fact, for this reaction, the basic strength was more important than mesoporosity. Mesoporous zeolites Y are also used as an ingredient of multi-component catalysts for fuel transformations, and they turned out to be superior to standard zeolites. Frequently, ^27^Al NMR signal from distorted tetrahedral Al is observed in mesoporous zeolites Y. The organization of zeolite crystals also gives an extremely porous system. It could be obtained by the crystallization of amorphous components or by optimization of hydrothermal treatment of zeolite Y.

## Figures and Tables

**Table 1:** The Si/Al values, the amounts of Si and Al extracted, N_2_ porosity, concentration of Brønsted and Lewis acid sites, the acid strength of Si-OH-Al groups ($∆\nu_{\mathrm{OH}\cdot \cdot \cdot \mathrm{CO}}$). Initial (after 1 min of reaction) conversion in α-pinene isomerization at 393 K. The data concerning parent zeolite Y (FAU-31) and zeolites desilicated by NaOH, TBAOH and NaOH/TBAOH mixture (10 mol % of TBAOH) at 293 K are presented. The data were adapted (with the permission) from [51].

Sample	Si/Al	% Extracted		Porosity cm^3^/g		Acidity			
						Concentration µmoles/g		$∆\nu_{\mathrm{OH}\cdot \cdot \cdot \mathrm{CO}}$ cm^−1^	Conversion %
		Si	Al	micro	meso	B.a.c.	L.a.c.		
FAU-31	31	-	-	0.33	0.20	282	86	354	12
NaOH	10.8	79	4.4	0.08	0.52	230	370	200	5
TBAOH	29.5	7	0.4	0.29	0.22	248	83	353	16
NaOH/TBAOH	17.3	43	4.2	0.21	0.89	310	160	354	53

**Table 2:** The Si/Al values, the amounts of Si and Al extracted, crystallinity, N_2_ porosity, concentration of Brønsted and Lewis acid sites, the acid strength of Si-OH-Al groups ($∆\nu_{\mathrm{OH}\cdot \cdot \cdot \mathrm{CO}}$). Conversion in α-pinene isomerization after 5 min of reaction at 363 K. The data concerning parent zeolite Y (FAU-31) and zeolites desilicated with NaOH/TBAOH mixtures of various TBAOH contents (0, 5, 10, 40, 70 and 100 mol % of TBAOH) are presented. The desilication was done at 293 K. The data were adapted (with the permission) from [53].

Sample	Si/Al	% Extracted		Crystallinity %	Porosity cm^3^/g		Acidity			Conversion %
							Concentration μmoles/g		$∆\nu_{\mathrm{OH}\cdot \cdot \cdot \mathrm{CO}}$ cm^−1^	
		Si	Al		micro	meso	B.a.c.	L.a.c.		
FAU-31	31	-	-	100	0.33	0.20	282	86	354	1
NaOH	10.8	78	7.8	0	0.08	0.52	230	370	200	1
5%	15.1	48	5.7	26	0.11	0.73	275	190	351	11
10%	17.3	43	4.2	50	0.21	0.89	310	160	353	14
40%	18.1	33	1.8	63	0.22	0.62	314	131	352	14
70%	21	25	1.7	78	0.25	0.63	315	116	351	12
100%	29.5	7	0.4	88	0.29	0.22	250	83	354	2

**Table 3:** The Si/Al values, the amounts of Si and Al extracted, crystallinity, N_2_ porosity, concentration of Brønsted and Lewis acid sites, the acid strength of Si-OH-Al groups ($∆\nu_{\mathrm{OH}\cdot \cdot \cdot \mathrm{CO}}$). Conversion in α-pinene isomerization after 5 min of reaction at 363 K. The data concerning parent zeolite Y (FAU-31) and zeolites desilicated with NaOH/TBAOH mixture at various temperatures (293, 318, 338, 353 and 373 K). The data were adapted (with the permission) from [53].

Sample	Si/Al	% Extracted		Crystallinity %	Porosity cm^3^/g		Acidity			Conversion %
							Concentration μmoles/g		$∆\nu_{\mathrm{OH}\cdot \cdot \cdot \mathrm{CO}}$ cm^−1^	
		Si	Al		micro	meso	B.a.c.	L.a.c.		
FAU-31	31	-	-	100	0.33	0.20	282	86	354	2
293 K	17.3	43	4.2	50	0.20	0.89	310	160	353	14
318 K	17.5	42	4.8	84	0.26	0.85	365	200	352	17
338 K	17.8	54	5.3	66	0.28	0.86	380	200	357	16
353 K	17.0	51	2.5	83	0.27	1.15	430	210	351	27
373 K	16.7	54	2.7	79	0.24	0.90	380	220	351	15

**Table 4:** The Si/Al values, the amounts of Si and Al extracted, N_2_ porosity, concentration of Brønsted and Lewis acid sites, the acid strength of Si-OH-Al groups ($∆\nu_{\mathrm{OH}\cdot \cdot \cdot \mathrm{CO}}$). Initial (after 1 min of reaction) at 393 K. The data concerning parent zeolite Y (FAU-31) and zeolites treated with 0.05 and 0.2 M solution of NH_3_ at 293 K. The data were adapted (with the permission) from [66].

Sample	Si/Al	% extracted		Porosity cm^3^/g		Acidity			Conversion %
						Concentration μmoles/g		$∆\nu_{\mathrm{OH}\cdot \cdot \cdot \mathrm{CO}}$ cm^−1^	
		Si	Al	micro	meso	B.a.c.	L.a.c.		
FAU-31	31			0.33	0.20	284	86	354	12
0.05 M NH_3_	31	1.8	0	0.09	0.41	182	155	352	21
0.2 M NH_3_	30	3.5	0	0.08	0.33	100	120	344	11

**Table 5:** The data on the acid strength of OH groups in zeolites: the frequency shifts of OH bands interacting with CO ($∆\nu_{\mathrm{OH}\cdot \cdot \cdot \mathrm{CO}}$) and with N_2_ ($∆\nu_{\mathrm{OH}\cdot \cdot \cdot N_{2}}$), the stretching frequency of CO molecule interacting with OH groups. The data were adapted (with the permission) from [52].

Zeolite	$\nu_{OH}\;{\mathrm{cm}}^{-1}$	$∆\nu_{OH\cdot \cdot \cdot CO}\;{\mathrm{cm}}^{-1}$	$∆\nu_{OH\cdot \cdot \cdot N_{2}}\;{\mathrm{cm}}^{-1}$	$\nu_{CO}\;{\mathrm{cm}}^{-1}$
ZSM-5	3615	315	132	2175
HY dealumin. (FAU-31)	3620	354	138	2180
HY desilicated	3626	354	138	2180
HY desilicated	3600	411	164	2183
USY	3600	380	143	2181
Mazzite dealuminated (Si/Al = 30)	3620	378	144	2180

## Abbreviations

B.a.c. – Brønsted acid centers; BEA – Beta zeolite; CTA^+^ - cetyltrimethylammonium cation; CTAB - cetyltrimethylammonium bromide; DFT – density functional theory; EFAL – extra framework aluminum; FCC – fluid catalytic cracking; FER – ferrierite; FT – fourier transform; IR - infrared spectroscopy; L.a.c. – Lewis acid centers; MAS – magic angle spinning; MAZ – mazzite; MD – molecular dynamics; MFI – Mobil Five; MOR – mordenite; NMR – nuclear magnetic resonance; PDA – pore-directing agents; Pluronic P123 - Poly(ethylene glycol)-block-poly(propylene glycol)-block-poly(ethylene glycol); RT – room temperature; TBA^+^ – tetrabutylammonium cation; TBABr - tetrabutylammonium bromide; TBAOH - tetrabutylammonium hydroxide; TEM – transmission electron microscope; TGA – thermogravimetric analysis; TMP – trimethylphoshpine; TMPO – trimethylphosphine oxide; TPA^+^ – tetrapropylammonium cation; TPAOH - tetrapropylammonium hydroxide; TPD – thermoprogrammed desorption; TPOAC - dimethyl octadecyl-(3-trimethoxysilylpropyl)-ammonium chloride; USY – ultra-stable zeolite Y; XRD – X-ray diffraction; XRF – X-ray fluorescence; ZSM-5 - Zeolite Socony Mobil–5
